# Platelet‐Released Growth Factors (PRGFs) Activate NRF2‐ARE and Modulate Inflammatory Response in an NRF2‐Dependent Manner in Primary Human Keratinocytes

**DOI:** 10.1111/jocd.70228

**Published:** 2025-05-12

**Authors:** Matthias Stein, Nicole Böttcher, Mersedeh Tohidnezhad, Athanassios Fragoulis, Andreas Bayer, Hannes Klump, Jens M. Baron, Thomas Pufe

**Affiliations:** ^1^ Department of Anatomy and Cell Biology Uniklinik RWTH Aachen University Aachen Germany; ^2^ Center for Clinical Anatomy Institute of Anatomy, Kiel University Kiel Germany; ^3^ Institute for Transfusion Medicine and Cell Therapeutics, Uniklinik RWTH Aachen University Aachen Germany; ^4^ Department of Dermatology and Allergology Uniklinik RWTH Aachen University Aachen Germany

**Keywords:** genetic analysis, inflammation, keratinocytes, NRF2, platelets, PRGF, skin barrier, skin physiology

## Abstract

**Background:**

Platelet‐released‐growth factors (PRGF) and platelet‐rich plasma (PRP) are blood‐derived products used in regenerative treatments and in overall aesthetic rejuvenation. Keratinocytes possess distinctive characteristics responsible for protection against environmental stressors and oxidant clearance. One such mechanism is the transcription factor NRF2, which plays a critical role in regulating cytoprotective genes, inflammation, and oxidative stress response. Data on the activation of the NRF2‐ARE and NF‐κB axes by PRGF are very limited.

**Aim:**

This study aims to investigate whether PRGF activates NRF2 and, if so, is responsible for the described anti‐inflammatory effect of PRGF/PRP in an in vitro primary human keratinocyte model.

**Methods:**

NRF2 activation is analyzed by NQO1 and HO‐1 western blotting, gene expression analysis, and by an ARE‐promoter study using luciferase‐based reporter gene assays in patient‐derived keratinocytes. Besides direct determination of the PRGF‐NRF2 interaction, we investigated the NF‐κB response by treating cells with PRGF and the inflammatory stimuli TNF‐α. Inflammatory parameters were analyzed using ELISAs for IL‐1β, IL‐4, Il‐10, TNF‐α and IL‐6 in the supernatant, NF‐κB luciferase reporter gene assays as well a‐NF‐κB western blotting. NRF2 involvement was tested by treating the cell‐culture model with the NRF2‐inhibitor ML‐385.

**Results:**

We were able to show that ARE activity was significantly upregulated in PRGF‐treated keratinocytes, leading subsequently to increased NQO1 and HO‐1 protein expression. Inflammatory IL‐secretion showed an association with NRF2 availability.

**Conclusions:**

In summary, PRGFs activate NRF2 target proteins and downregulate NF‐κB‐associated inflammation in an NRF2‐dependent manner. Therefore, we further suggest PRGF as an anti‐inflammatory treatment after medical aesthetic procedures.

## 1 Introduction

Homologous platelet products such as platelet‐rich plasma (PRP) and platelet‐released growth factors (PRGFs) exhibit significant advantages in regeneration processes and serve as treatments for chronic wounds and regenerative defects. The primary mechanism of action is the release of multiple growth factors such as epidermal growth factor (EGF), vascular endothelial growth factor (VEGF), platelet‐derived growth factor (PDGF), fibroblast growth factor (FGF), insulin‐like growth factor (IGF), and other chemokines, which orchestrate proliferation, migration, and inflammatory response and thus exhibit strong tissue‐regenerative potential [1]. PRGF is a cell‐, plasma‐, and fibrin‐free lysate of thrombocytes that lacks the viscoelastic effect of activated PRP or platelet‐rich fibrin (PRF). While PRF‐induced coagulation and clot formation have been reported to be beneficial for treating macroscopic, viscoelastic‐dependent cartilage defects or bone fractures, it limits PRP and PRF application in more delicate structures such as macular degeneration or in small‐volume subcutaneous injections [2, 3, 4]. One study even reported the necessity of micronizing PRP clots to ensure optimum growth factor release and overcome injection issues caused by coagulation [5]. PRP and PRGF‐like formulations are well established in dental surgical procedures to improve gingival recovery from flap surgeries and implant‐to‐bone acceptance and osteogenesis [6, 7]. Certain PRGF preparations potentiate growth‐factor levels up to 20 times the basal level without gelation, cell residual contamination, or use of anticoagulants [7, 8]. This makes PRGF a blendable stimulant for studying its in vitro properties while preventing unwanted viscosity‐dependent background effects on cells. PRGF induces extracellular matrix gene expression and keratinocyte cell differentiation [8, 9]. Moreover, it was demonstrated that PRGF induces nuclear factor erythroid 2‐related factor 2 (NRF2) release and expression of target genes such as NAD(P)H quinone dehydrogenase1 (NQO1) and heme‐oxygenase 1 (HO‐1), resulting in cryoprotection and regeneration of human osteoblasts, tenocytes, and retina cells [4, 10, 11, 12].

NRF2 is a key regulator of oxidative stress response, cellular protection, epithelial repair, detoxification, and redox homeostasis in keratinocytes [13, 14]. Under basal conditions, NRF2 is bound via cysteine residue interactions to its repressor protein, Kelch‐like ECH‐associated protein 1 (KEAP1), which mediates the rapid proteasomal degradation of NRF2 [15]. NRF2 degradation is also regulated by the non‐canonical pathway involving glycogen synthase kinase 3 beta (GSK3‐β) downstream processes [16]. In response to endogenous and exogenous stressors such as oxidants or electrophiles, the NRF2‐KEAP1 complex is disrupted, releases NRF2 to translocate to the nucleus, and activates antioxidant response element (ARE)‐containing target‐gene expression. Further activation of NRF2 is achieved by growth factor‐mediated inhibition of GSK3‐β [17, 18]. Under physiological conditions, small amounts of NRF2 escape from KEAP1 and induce basal target‐gene expression. NRF2‐stimulating compounds like sulforaphane (SFN) or methysticin (MET) demonstrate electrophilic potential, resulting in rapid KEAP1 conformational changes followed by NRF2 activation [19, 20, 21]. NRF2 targets genes such as NQO1, HO‐1, thioredoxin reductase 1 (TXNRD1), and glutathione S‐transferase (GST), which drive antioxidant clearance and thereby reduce oxidative stress. However, it should be mentioned that NQO1 is the only direct target of activated NRF2 [13, 22, 23].

Most wound inflammasomes are characterized by increased oxidative stress leading to inhibited keratinocyte and fibroblast interaction and an interleukin imbalance, which may prevents regeneration and the functioning of physiological‐barrier processes in the skin [24]. Activation of NRF2 in the dermis has been linked to improved wound closure as well as enhanced epithelial repair in diabetic rodents [25]. Interestingly, pharmacological NRF2 activation leads to increased keratinocyte proliferation and re‐epithelialization, induced by a senescent fibroblast secretome rich in growth factors in vivo [26]. NRF2 stimulation in primary human keratinocytes showed that NRF2 activators target inflammatory clearance indirectly by inhibiting NF‐κB [27].

Reduced NRF2 activity and increased oxidant levels are also observed in patients with lesional and regeneration defective wound phenotypes, suggesting that activating NRF2 could be a promising target for clinical treatments [28, 29, 30]. Wounds show an imbalance between the release and degradation of collagens and growth factors, resulting in wound inflammation that may disrupt the physiological wound microbiome and defense [31, 32, 33].

Extrapolating from previous studies, we hypothesized that PRGF exhibits an NRF2‐ARE activating effect on primary normal human epidermal keratinocytes (NHEKs) that downregulates inflammation in an NRF2‐dependent manner.

## 2 Methods

### 2.1 Cell Culture

Primary keratinocytes were isolated from foreskin tissue obtained from six patients, aged 5–17, who underwent urological surgery and agreed to the use of their cells for research purposes. NHEKs were cultured in monolayers using a 1:1 serum‐free mixture of high‐glucose (4.5 g/L) Dulbecco's Modified Eagle's Medium (DMEM) and DermaLife K Keratinocyte Growth Medium supplemented with selected LifeFactors (LifeLine LL‐0077) and Gibco GlutaMAX 100X (ThermoFisher Scientific). All media were supplemented with penicillin–streptomycin (100 IU/mL penicillin, 0.1 mg/mL streptomycin). Cells were cultured on collagen‐A (0.1 mg/mL) (Biochrom GmbH, Germany) coated surfaces in a humidified atmosphere with 5% (v/v) carbon dioxide at 37°C. Prior to stimulation, cells were starved to minimize proliferation while maintaining significant cell viability. NHEKs from passages 1–3 were used for the experiments. Cell morphology was constantly monitored using manual light microscopy (DM IL, Leica Microsystems GmbH, Wetzlar).

### 2.2 PRGF Preparation

Donors gave their written consent for their use in research activity according to the Ethics Board EK116/10.

Thirty platelet concentrates from male and female donors aged 18–62 years were collected, pooled, and lysed by repetitive freeze–thaw cycles, as described previously [10]. PRGF aliquots were normalized to ~3.0 × 10^6^ platelets/μL to yield a 10‐fold platelet concentration. Leucocyte concentration was determined to be below 5 × 10^4^ using a hemocytometer.

PRGFs were stored at −80°C and used immediately after the final thawing step for stimulation.

### 2.3 Cell Viability and Proliferation Assay

To investigate the effect of PRGF and SFN (Sigma Aldrich, #S6317) on cell viability and proliferation, cells were treated with 5%, 10%, and 15% PRGF and 5, 10, and 15 μM SFN for 24 h against a DMSO vehicle control.

Cell viability was assessed using the Cell‐Titer‐Blue (CTB) Viability Assay (Promega, USA, #G8080) according to the manufacturer's instructions. Briefly, viable cells reduce the CTB dye resazurin to resorufin, which emits fluorescence at 590 nm. The emitted fluorescence is proportional to cellular metabolism and therefore viability. Cells were incubated for 1 h at 37°C with the CTB dye and immediately measured with a fluorescence microplate reader (TECAN, Switzerland, Infinite M200).

The CyQuant Cell Proliferation Assay Kit (Invitrogen, USA, #C7026) was used to determine total cell count, according to the manufacturer's instructions. The CyQuant GR Dye shows intense fluorescent emission at 520 nm when bound to DNA and provides linear detection within cell populations that is independent of cell metabolism. Therefore, cell culture media was removed and replaced with CyQuant lysis buffer, frozen and thawed, mixed with the CyQuant GR Dye, and immediately measured with a fluorescence microplate reader (TECAN, Switzerland, Infinite M200). Prior to stimulation, cells were starved to ensure a low proliferative state and moderate viability to investigate the influence of the stimulants PRGF and SFN.

### 2.4 Western Blot Analysis

The steady‐state amount of NQO1 and HO‐1 protein was assessed by western blotting of whole cell lysates. NHEKs were treated with 10 μM SFN as a positive control, 5% and 10% PRGF compared to an untreated group and DMSO vehicle group. Inflammatory response was assessed by western blotting whole cell lysates for NFκB‐p65 and phosphorylated (activated) NFκB‐p65 (p‐NFκB) after PRGF, SFN, and TNF‐α (co)‐stimulation. Cells were seeded in 10 cm petri dishes at a density of 5 × 10^5^ cells/cm^2^. Upon confluency, cells were stimulated for 24 h. Cell lysis and sample preparation followed the standard protocol provided by Abcam (ab152163, UK, Abcam, 2020).

Protein concentration was determined using the PierceTM BCA Protein Assay Kit (Thermo Fisher Scientific, USA #23225) and a fluorescence microplate reader (TECAN, Switzerland, Infinite M200). Western blot samples were prepared by mixing ultrapure water, dithiothreitol, and NuPAGE LDS sample buffer (Invitrogen, USA, #NP0007), and exposing them to heat at 95°C for 35 min. Eight micrograms of protein per pocket were loaded onto a 12% SDS‐PAGE gel and separated by electrophoresis. Proteins were transferred onto a PVDF membrane and blocked for 1 h with 5% milk in Tris‐buffered saline with Tween (TBS‐T). The NQO1 primary antibody (Abcam UK, #80588, 1:1000, diluted in 5% milk TBS‐T), HO‐1 primary antibody (Santa Cruz, #J1112, 1:1000, diluted in 5% milk TBS‐T), NF‐κB p65 primary antibody (Cell Signaling Technology, USA, #4764, 1:1000, diluted in 5% milk TBS‐T), and p‐NF‐κB‐p65 (Ser 536) (Cell Signaling Technology, USA, #3031, 1:1000, diluted in 5% milk TBS‐T) primary antibody were incubated for 1 h at room temperature. The secondary HRP‐conjugated antibodies (Sigma‐Aldrich, USA, #A4416 and Santa Cruz, #B2516, diluted 1:500, diluted in TBS‐T) were incubated for 1 h at room temperature. Chemiluminescence was detected using WESTAR SUPERNOVA (CYANAGEN, Srl Italia, #XLS3, 0100) and visualized with Fusion Solo X (Vilber, Germany). Analysis was performed using ImageJ 1.54d (National Institutes of Health, USA) with α‐tubulin as the reference protein. α‐Tubulin detection was achieved after heat stripping, blocking, and overnight incubation with α‐tubulin primary antibody (Santa Cruz, USA, #sc‐5286, 1:1000, diluted in 5% milk TBS‐T), followed by secondary anti‐mouse antibody (Sigma‐Aldrich, USA, #A4416, 1:500, diluted in TBS‐T).

### 2.5 Gene Expression Analysis

Keratinocytes were seeded and stimulated as described before, and RNA was extracted using RNA‐Solv Reagent (Omega Bio‐Tek Inc., USA). RNA levels and purity were analyzed spectrophotometrically using a NanoDrop 1000 Spectrophotometer (Thermo Fisher Scientific, USA).

DNase I (Ambion, Germany, #AM222) and 2 μq of sample RNA were used for DNA digestion according to the manufacturer's instructions. The cDNA was synthesized using Maxima Reverse Transcriptase (Thermo Fisher Scientific, USA, #EPO742) with mixed priming [0.05 μg random hexamer, 0.375 μg oligo‐(dT)18, and 1 μL dNTP mix (10 mM each) (Thermo Fisher Scientific, USA, #SO132, #SO142, #R0192)].

The Reverse Transcriptase quantitative polymerase chain reaction (RT‐qPCR) was done on an ABI StepOne Plus system (Thermo Fisher Scientific, USA) using the PowerSYBR Green PCR Master Mix (Thermo Fisher Scientific, USA). Primer Annealing temperatures were pre‐evaluated for each primer. Melting curves were analyzed to secure primer specificity. The LinRegPCR analysis 2021.2 (J.M. Ruijter, The Netherlands) based on Ruijter et al., was used to calculate individual amplification efficiencies [34]. The mentioned reference genes were pre‐evaluated for this study using the qbase+ software (Biogazelle, Belgium). Briefly, it was screened for 10 potential reference genes in 10 representative samples to allow data normalization. For our study, human ribosomal protein L13 (hRPL13) and human ribosomal protein S6 (hRPS6) were selected as reference genes. Relative fold of mRNA expression was calculated with the qbase+ software, applying the efficiency‐corrected ΔΔCq method described by M.W. Pfaffl [35]. The qPCR primer list is shown in Table 1.

**TABLE 1:** All used primer pairs for qPCR.

Target type	Gene	Sequence accession number (LRG)	Direction	Sequence	Annealing temperature	Melting temperature
Target gene	NQO1	NM_001286137.2	Forward Reverse	TGCAGCGGCTTTGAAGAAGA TCCTTCAGTTTACCTGTGATGTCC	62.5°C	60.5°C 60.3°C
	HO‐1	NM_002133.3	Forward Reverse	TGACCCATGACACCAAGGAC AGTGTAAGGACCCATCGGAGA	59.0°C	59.6°C 60.0°C
Reference gene	SDHA	NM_004168	Forward Reverse	TGGAACAAGAGGGCATCTG CCACCACTGCATCAAATTCATG	61.5°C	59.4°C 58.4°C
	ACTB	NM_001101.3	Forward Reverse	CTGGAACGGTGAAGGTGACA AAGGGACTTCCTGTAACAACGCA	61.5°C	59.4°C 58.9°C
	B2M	NM_004048	Forward Reverse	TGCTGTCTCCATGTTTGATGTATCT TCTCTGCTCCCCACCTCTAAGT	62.5°C	59.7°C 62.1°C
	GAPDH	NM_002046.5	Forward Reverse	TGCACCACCAACTGCTTAGC GGCATGGACTGTGGTCATGAG	61.5°C	59.4°C 61.8°C
	GUSB	NM_000181.3	Forward Reverse	GGACCGGGAAGCATGGCT GGGGCCTGACTCCCACA	60.5°C	60.5°C 60.6°C
	HPRT1	NM_000194	Forward Reverse	TGACACTGGCAAAACAATGCA GGTCCTTTTCACCAGCAAGCT	61.5°C	55.9°C 59.8°C
	YWHAZ	NM_003406	Forward Reverse	ACTTTTGGTACATTGTGGCTTCAA CCGCCAGGACAAACCAGTAT	61.5°C	57.6°C 59.4°C
	RPL13A	NM_012423	Forward Reverse	CCTGGAGGAGAAGAGGAAAGAGA TTGAGGACCTCTGTGTATTTGTCAA	60.5°C	62.4°C 59.7°C
	RPS6	NM_001010.2	Forward Reverse	TGATGTCCGCCAGTATGTTGT CTTAGCCTCCTTCATTCTCTTGG	59.0°C	59.7°C 58.6°C
	SDHA	NM_004168	Forward Reverse	TGGAACAAGAGGGCATCTG CCACCACTGCATCAAATTCATG	61.5°C	59.4°C 58.4°C

### 2.6 Luciferase‐Based Reporter Gene Assays (ARE‐Luc and NFκB‐Luc)

NRF2‐ARE activation and NF‐κB‐dependent inflammatory signaling were assessed using luciferase‐based reporter cell lines and assays (Luciferase Assay Systems Kit, Promega, USA, #E1500) to identify optimum stimulation concentrations and promoter response.

Stably transfected NHEK SIN‐lenti‐ARE and NHEK SIN‐lenti‐NF‐κB cells were generated with lentiviral particle transduction as previously described [36]. Promoter activity was determined by luminescent detection, and luminescent signals were normalized to a fluorescence‐dependent total cell numbers using the CyQuant Assay ([luminescence signal]/[fluorescence signal]) and a fluorescence microplate reader (TECAN, Switzerland, Infinite M200).

Transduced cells were seeded at a density of 5 × 10^4^ cells/cm^2^ in a 24‐well plate and cultivated until confluency. To investigate ARE activation and study concentrations, cells were stimulated with 10 μM SFN, 10% PRGF, DMSO, and 10 ng/mL TNF‐α. To study NF‐κB response on inflammatory stress, NHEK SIN‐lenti‐NF‐κB cells were exposed to co‐stimulation using 10 ng/mL TNF‐α for 6 h, followed by SFN and PRGF for 24 h. In reverse, NHEK sin‐lenti‐NF‐κB cells were pre‐conditioned with SFN and PRGF for 24 h followed by a 6‐h 10 ng/mL TNF‐α co‐stimulation.

NRF2‐dependent luciferase‐indicated promoter activity was inhibited for both cell lines using 5 μM ML‐385 (NRF2 Inhibitor: N‐[4‐[2,3‐Dihydro‐1‐(2‐methylbenzoyl)‐1H‐indol‐5‐yl]‐5‐methyl‐2‐thiazolyl]‐1,3‐benzodioxole‐5‐acetamide) (#6243/5, R&D Systems) for 3–4 days prior to stimulation to ensure sufficient NRF2 inhibition.

### 2.7 Enzyme‐Linked Immunosorbent Assay

Post‐stimulation quantification of secreted pro‐ and anti‐inflammatory cytokines was performed using commercially available ELISA kits. Cells were cultivated and (co)‐stimulated as described in section 2.5. Supernatants were collected after cells were washed and incubated with culture medium for 24 h after stimulation. IL‐1β (R&D Systems, DY201), IL‐6 (R&D Systems, DY206), IL‐10 (PeproTech, 900‐K21), IL‐4 (PeproTech, 900‐T14) and TNF‐α (R&D Systems, DY210) ELISA kits were used according to the manufacturers' instructions and measured using a fluorescence microplate reader (TECAN, Switzerland, Infinite M200). Total cell numbers were assessed by the CyQuant assay as described previously.

PRGF samples were characterized using the following ELISA kits: PDGF‐BB (R&D systems, DY220) and EGF (PeproTech, 900‐K05) as well as IL‐1β, IL‐6, IL‐10, TNF‐α and IL‐4. PRGF samples were normalized to platelet count and volume to quantify IL content/mL.

### 2.8 Statistics

All experiments were done in triplicates, at least. Statistical analysis was done using GraphPad Prism 9.5.0 (GraphPad, USA). One‐way ANOVA and Dunnett's multiple comparisons test were used to check parametric data. Column and row effects were investigated with two‐way ANOVA and Tukey's multiple comparison post hoc test. The Shapiro–Wilk test was used to investigate the normal distribution of the data, while the Bartlett's test was used to test for heteroscedasticity. Statistical significance is indicated by *p < 0.05; ****p < 0.0001, and all data are shown as the arithmetic mean ± standard deviation (SD).

## 3 Results

### 3.1 Effect of PRGF on Keratinocyte Viability and Differentiation

The combination of both CTB and CyQuant assay allowed to investigate the biological activity of PRGF and SFN on keratinocyte viability and proliferation.

**FIGURE 1:**
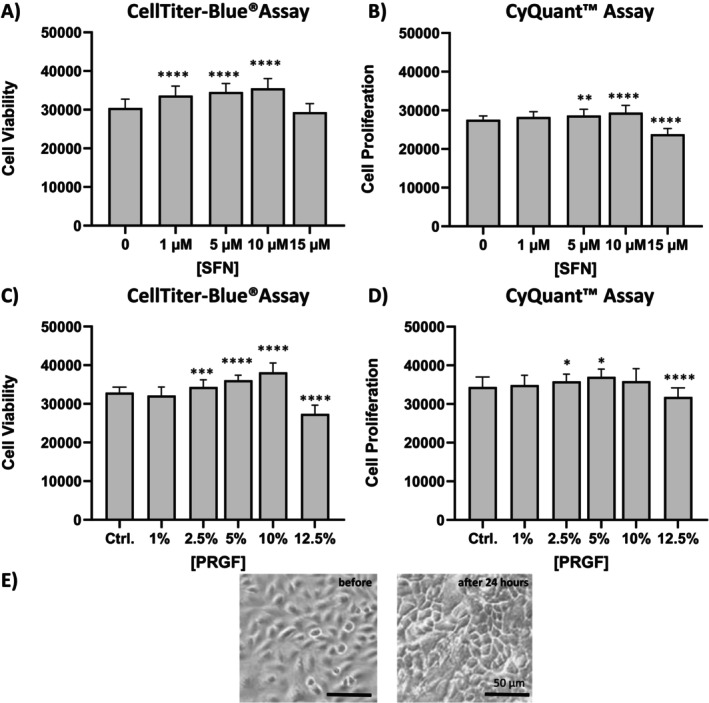
Viability and cell proliferation of SFN (A, B) and PRGF (C, D)‐treated NHEKs. Cell viability was measured using the CTB assay (Ex560/Em590). The resazurin value of the control group was used to compare measurements. Cell proliferation was assessed by determining DNA‐contents using the CyQuant assay, and compared to control groups (Ex^480^/Em^520^). Data are shown as a mean ± SD; statistical significance is indicated with *p < 0.05, **p < 0.005, ***p < 0.001 and ****p < 0.0001 versus the control group (*n* = 6). (D) PRGF induces a terminal differentiated morphology in NHEKs after 24 h; scale bar = 50 μm.

Stimulation with 1, 5, and 10 μM SFN led to significantly increased cell viability compared to untreated cells, while reduced viability was detected in concentrations exceeding 10 μM SFN (Figure 1A,B). PRGF exposure increased keratinocyte viability for 2.5%, 5%, and 10% PRGF (Figure 1C,D), while increased cell proliferation was limited to 2.5% and 5% PRGF, indicating the highest cell viability for 10% PRGF while inhibiting cell proliferation (Figure 1D). All data were compared against an unstimulated control group. Light microscopy revealed that 10% PRGF induces a terminally differentiated keratinocyte morphology (Figure 1E). Therefore, study concentrations were set to 5%–10% PRGF and 10 μM SFN.

### 3.2 PRGF Induces ARE Activity and Stimulates NQO1 and HO‐1 Expression

**FIGURE 2:**
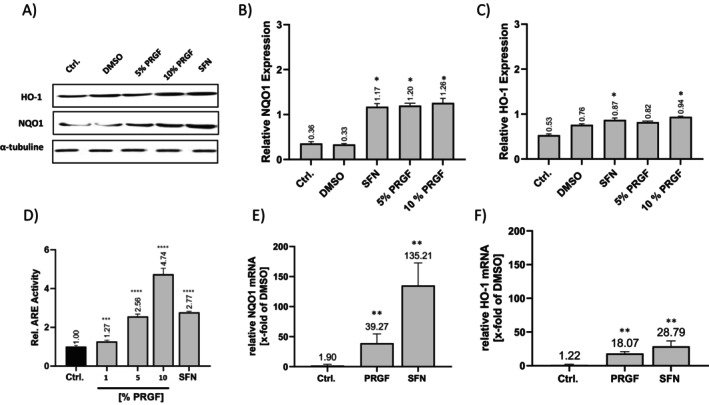
PRGF‐induces (A–C) NQO1 and HO‐1 protein expression, (D) ARE promoter activity and (E, F) NRF2 target gene expression. (A) Cells were stimulated and subjected to (A) western blotting using specific (B) NQO1, (C) HO‐1 and α‐tubuline antibodies. (D) Transduced NHEKs were subjected to mono‐luciferase assay and CyQuant assay after stimulation to calculate relative ARE‐activity. The luminescence values of the experimental group treated with DMSO vehicle control (Ctrl.) were used only as a control and were set to 1. Gene expression analysis of (E) NQO1 and (F) HO‐1 was investigated by RT‐qPCR. Data were expressed relative to the vehicle control. Data are shown as the mean ± SD; statistical significance is indicated in (A) with *p < 0.05, (*n* = 3), (B) ***p < 0.0005, ****p < 0.0001, (*n* = 7) and (C) with **p < 0.05, (*n* = 5) versus the respective control group.

10% PRGF and SFN‐stimulated NHEKs showed a significant increase in protein expression of the NRF2 targets NQO1 and HO‐1 compared to the control and DMSO groups, indicating NRF2 activation after 24 h of stimulation. Further, as a result of NRF2 activation, NQO1xpression was also significantly upregulated by 5% PRGF stimulation. SFN was used as a positive control (Figure 2). All stimulations led to a relative increase in ARE activity; the relative ARE activity of PRGF in comparison to SFN and the control group showed a 4.74‐fold ±0.28 increase for 10% PRGF, while 10 μM sulforaphane leads to a 2.77‐fold ±0.05 increase in ARE activity. 1% and 5% PRGF increased relative ARE activity respectively to 1.27 ± 0.07 and 2.56 ± 0.10 (Figure 2D). Further, mRNA expression of NRF2 target genes NQO1 and HO‐1 was observed using RT‐qPCR. The NQO1 mRNA expression increased 39.27‐fold, while HO‐1 mRNA expression increased 18.07‐fold as a result of PRGF stimulation. The NRF2 stimulant SFN led to a 135.21‐fold increase in NQO1 and a 28.79‐fold increase for HO‐1 compared to the vehicle group.

**FIGURE 3:**
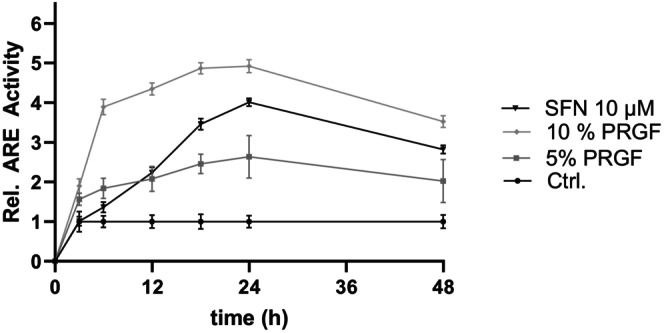
Time and stimulation dependent ARE promoter kinetics mediated by SFN and PRGF. NHEK SIN‐lenti‐ARE cells were stimulated with PRGF (5% and 10%) and 10 μM SFN for 48 h (3, 6, 12, 18, 24 and 48 h) and analyzed as described earlier (section 3.2). Data are shown as the mean ± SD (*n* = 6) and found to be statistically significant (****p < 0.0001; not shown for readability reasons) against the control group.

**FIGURE 4:**
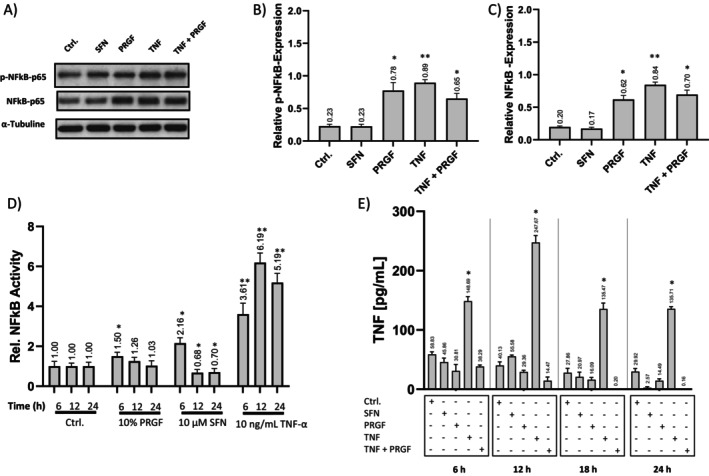
NF‐κB response was suppressed by PRGF‐NRF2‐activating treatment. (A) Cells were stimulated and subjected to western blotting using specific (B) NF‐κB‐p65, (C) p‐NF‐κB‐p65 (Ser 536) and α‐tubulin antibodies (*n* = 3). (D) NHEK sin‐lenti‐NF‐κB‐Luc cells were pre‐conditioned with PRGF (5%, 10% PRGF) or 10 μM SFN for 6, 12, and 24 h, followed by media exchange and immediate co‐stimulation with 10 ng/mL TNF‐α for another 6 h. The luminescence values of the experimental group (treated with DMSO) were used only as a control and were set to 1. Single stimulation with 10 ng/mL TNF‐α without NRF2 activating pre‐conditioning was used as a negative control (*n* = 6). (E) Keratinocytes were stimulated with 10 μM SFN, 10% PRGF, 10 ng/mL TNF‐α and co‐stimulated with 10 ng/mL TNF‐α + 10% PRGF for various time points. Supernatants were subjected to TNF‐α‐ELISA. Data are shown as the mean ± SD; statistical significance is indicated at *p < 0.05 and **p < 0.005 versus the control group.

Stimulation‐time‐dependent ARE activity was investigated for 48 h and assessed at various points in time. Relative ARE activity was significantly increased for PRGF and SFN, reaching peak activity at 24 h; however, PRGF showed a faster initial response after 6 h. The delay in response to SFN treatment is shown in Figure 4. A dose‐dependent increase in ARE activity was detectable at 3 h (for 10% PRGF: 1.9‐fold ±0.18; for 5% PRGF: 1.56‐fold ±0.15), and it peaked at 24 h (for 10% PRGF: 4.9‐fold ±0.17; 5% PRGF: 2.6‐fold ±0.54; SFN: 4.0‐fold ±0.1) (Figure 3). Study parameters were set to 10% PRGF and 10 μM SFN, as a positive control, to achieve highest ARE activity.

### 3.3 PRGF Alleviates NF‐κB Keratinocyte Activation

The effect of PRGF and SFN stimulation was investigated by NF‐κB‐p65 and p‐NF‐κB‐p65 (Ser 536) western blotting of keratinocyte whole cell lysates. Stimulation with SFN alone led to no significant increase of NF‐κB‐p65 and p‐NF‐κB‐p65 compared to the control group. The stimulation groups treated with TNF‐α, as well as the co‐stimulation group treated with TNF‐α and PRGF, showed significantly increased protein levels of NF‐κB‐p65 and p‐NF‐κB‐p65. Moreover, the co‐stimulation with TNF‐α and PRGF resulted in a lower signal for both markers. PRGF treatment led to increased levels of NF‐κB‐p65 and p‐NF‐κB‐p65 compared to the control group.

Luciferase based NF‐κB response was investigated by pre‐treatment of NHEK SIN‐lenti NF‐κB cells with PRGF and SFN prior to TNF‐α stimulation. All PRGF and SFN pre‐conditioned groups showed reduced NF‐κB reactivity upon TNF‐α inflammatory stress compared to TNF‐α stimulation alone. Twelve‐ and 24‐h SFN pre‐stimulation even suppress NF‐κB signaling, and 6‐h pre‐stimulation showed a 2.16‐fold increase. A single TNF‐α stimulation led to a 3.61‐ to 6.19‐fold increase of NF‐κB signals (Figure 4B). Like SFN, stimulation with 10% PRGF promotes NF‐κB resilience from 6 to 24 h. Next to protein expression and promoter activity, the activation of NF‐κB by the stimulation was investigated by using TNF‐α ELISA of the supernatants for a time course of 24 h. The TNF‐α secretion post stimulation was significantly increased for all study groups. Interestingly, TNF‐α levels peaked in the 12 h group upon TNF‐α stimulation. Further, the TNF‐α levels in NRF2 activating treatments were the lowest and decreased over the 24 h time course, indicating sufficient inhibition of the NF‐κB pathway.

### 3.4 NF‐κB‐ and ARE‐Promoter Activity of PRGF Is NRF2‐Dependent

We observed a significantly increased ARE‐activity in comparison to the vehicle control group after SFN (+2.02‐fold), PRGF (+3.68‐fold), and TNF‐α (+5.36‐fold) as well as the TNF‐α–PRGF co‐stimulation (+4.60‐fold). Addition of ML‐385 almost abrogated responses to SFN (+1.19‐fold), PRGF (+1.07‐fold), TNF (+1.66‐fold) and TNF‐α–PRGF (+2.11‐fold). These results demonstrate an effective inhibition of NRF2 in vitro, according to the reduced ARE activation of the positive control SFN and, vice versa, proof PRGF as an NRF2 activator in primary keratinocytes.

In addition, investigation of NF‐κB activity showed a reduced response for SFN (0.77‐fold), PRGF (0.74‐fold), and TNF‐α–PRGF co‐stimulation (0.79‐fold). TNF‐α stimulation alone increased NF‐κB signaling 9.87‐fold compared to the vehicle control under non‐inhibited conditions. Inhibition of NRF2 resulted in an overall increase NF‐κB activation for SFN (1.27‐fold), PRGF (1.02‐fold), TNF‐α (8.53‐fold), and for TNF‐α–PRGF co‐stimulation (6.94‐fold). As previously observed, the PRGF‐inflammatory rescue effect on the TNF‐α‐activated NF‐κB response completely reversed under NRF2 inhibition, highlighting that this effect is NRF2‐mediated.

**FIGURE 5:**
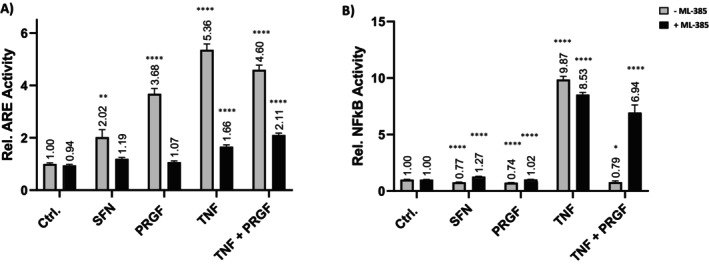
Effect of ML‐385‐mediated NRF2 inhibition on (A) ARE‐Luc and (B) NF‐κB‐Luc reactivity in stimulated NHEKs. Cells were subjected to luciferase and CyQuant assays after stimulation to estimate the relative promoter activities. NRF2 inhibition led to an overall reduction of ARE activity and elevated NF‐κB response compared to unblocked NRF2. Data are shown as the mean ± SD; statistical significance is indicated at *p < 0.05 and ****p < 0.0001 versus the control group without ML‐385 (*n* = 6).

Combining the data from the promoter study, we found that PRGF stimulates NRF2‐ARE and down‐regulates a TNF‐α‐associated inflammatory stress response that is NRF2‐mediated (Figure 5).

### 3.5 PRGF Influences Pro‐ and Anti‐Inflammatory Cytokine Secretion in an NRF2‐Dependent Manner

NRF2 activators are known for their anti‐inflammatory and cytokine‐modulatory effects. Thus, ML‐385 treated keratinocyte supernatants were assayed by ELISA to investigate cytokine release post‐stimulation.

TNF‐α treatment resulted in IL‐1β (508.4 pg/mL ± 11.8) and IL‐6 (1196.0 pg/mL ± 216.7) secretion compared to the control group, which could not be eliminated in PRGF co‐stimulation (IL‐1β: 492.6 pg/mL ± 6.6; IL‐6: 1150.9 pg/mL ± 54.7) under ML‐385 inhibitory conditions. Without ML‐385, cells showed a TNF‐α response (IL‐1β: 461.57 pg/mL ± 10.7; IL‐6: 1251 pg/mL ± 48.7) that was significantly reduced with PRGF co‐stimulation (IL‐1β: 176.6 pg/mL ± 2.4; IL‐6: 803.0 pg/mL ± 27.8).

**FIGURE 6:**
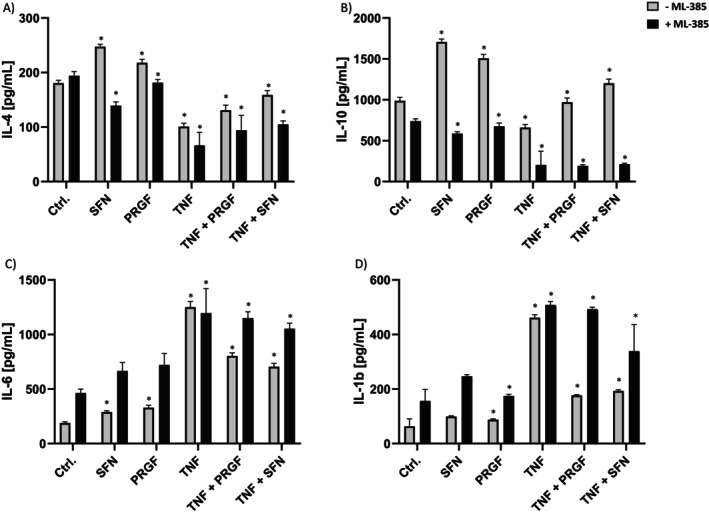
Effect of ML‐385‐mediated NRF2 inhibition on NHEKs cytokine release. Secretion levels of (A) IL‐4, (B) IL‐10, (C) IL‐6 and (D) IL‐1β in the supernatants were analyzed using ELISA kits. NRF2 inhibition was mediated by using 5 μM ML‐385 (black bars). Data are shown as the mean ± SD; statistical significance is indicated at *p < 0.05 versus the control group (*n* = 6).

PRGF treatment mediated an increase of anti‐inflammatory cytokines IL‐4 (247.4 pg/mL ± 4.3) and IL‐10 (1707.6 pg/mL ± 32.9) release, which reversed under NRF2 inhibition to IL‐4 (139.5 pg/mL ± 6.5) and IL‐10 (586.8 pg/mL ± 20.9) compared to the respective control groups (Figure 6). Moreover, PRGF treatment led to a downregulation of pro‐inflammatory IL‐1β (99.26 pg/mL ± 2.3) and IL‐6 (289.86 pg/mL ± 10.5) compared to the TNF‐α group, but not compared to the untreated control group. NRF2 inhibition resulted in a generally increased pro‐inflammatory cytokine release that could not be rescued with PRGF treatment for IL‐1β (246.8 pg/mL ± 5.8) and IL‐6 (665.0 pg/mL ± 75.3) compared to the control group (IL‐1β: 156.12 pg/mL ± 40.3; IL‐6: 463.5 pg/mL ± 34.1).

Co‐stimulation with TNF‐α and PRGF led to increased IL‐4 (131 pg/mL ± 8.8) and IL‐10 (971.1 pg/mL ± 49.3) levels compared to the TNF‐α treatment alone (IL‐4: 100.8 pg/mL ± 8.8; IL‐10: 660.4 pg/mL ± 36.4), but not compared to the untreated control group (IL‐4: 180.7 pg/mL ± 5.0; IL‐10: 987.04 pg/mL ± 40.5). NRF2 inhibition mediated a reduction in IL‐4 and IL‐10 cytokine release for PRGF and SFN single treatments, but not for the untreated control group; however, TNF‐α treatment caused a reduction in IL‐4 (66.1 pg/mL ± 23.2) and IL‐10 (153.2 pg/mL ± 13.81) secretion, which, similar to the non–ML‐385 treatment, was reversed with PRGF co‐stimulation (IL‐4: 94.1 pg/mL ± 26.2; IL‐10: 192.0 pg/mL ± 14.3).

PRGF content was investigated for the following analytes: PDGF‐BB, EGF, IL‐4, IL‐6, IL‐8, IL‐10 and S100‐protein ELISA and found to be consistent and reproducible over the study period and beyond, indicating PDGF‐BB (3316.4 pg/mL ± 193.15 pg/mL), EGF (725.7 pg/mL ± 232.4 pg/mL) and IL‐4 (803 pg/mL ± 82.3 pg/mL) as the most abundant actives, while IL‐10 (208.2 pg/mL), S100 (195.6 pg/mL), IL‐1β (144.2 pg/mL), IL‐8 (33.1 pg/mL) and IL‐6 (9.0 pg/mL) represented the minor fractions (see Figure S1).

## 4 Discussion

Activation of the transcription factor NRF2 goes hand in hand with multiple biochemical mechanisms, mainly involved in clearing oxidative stress and regulating redox homeostasis. Keratinocytes are constantly exposed to environmental stressors, indicating NRF2 as a key transcription factor for defense mechanisms and terrestrial damage prevention. While normal cells tolerate a broad range of stressors, mechanically stressed or pathologic conditions respond with severe disequilibrium, resulting in inflammation, reduced wound healing, or even infections, all of which involve NRF2 activation or deactivation [27, 33].

In this study, primary human keratinocytes under physiological and inflammatory conditions were treated with PRGF to investigate and pinpoint the activation and involvement of NRF2 in the anti‐inflammatory effect of platelet‐derived mediators. To the best of our knowledge, no available in vitro or clinical evidence is available that connects the benefits of NF‐κB downregulation of PRGF with the activation and involvement of NRF2.

The activity of PRP/PRGFs from individual donors varies per se and is thus a strongly influential factor in in vitro and in vivo studies. Our protocol aims to diminish variations in donor and preparation procedures by normalizing the platelet count of apheresis thrombocyte concentrates. For transparency and reproducibility reasons, we introduce the PRP code 110–01–10 according to Kon et al. to describe our PRGF [37]. PRGF analysis revealed the presence of multiple cytokines and growth factors such as PDGF‐BB, IL‐4, EGF, IL‐10, S100 proteins, IL1b, IL‐8, TNF‐α and IL‐6. Previously published studies showed that EGF receptor activation mediates NRF2 activation that also upregulates EGF‐ and PDGF‐receptor ligands resulting in further growth‐factor release. PDGF‐BB has been reported to activate NRF2‐ARE gene expression in a rodent renal cell culture model [38, 39]. Additionally, IL‐4 is reported to induce NRF2 and to drive cellular regeneration in retinal pigment epithelium cells [40]. S100 protein members such as S100A7 (psoriasin) have also been reported to increase NRF2‐ARE activation as well [41]. These findings are broadly consistent with the data of our stimulation model and underline the observed NRF2‐ARE activity mediated by PRGFs.

Compared to commercial and recombinant growth factor kits or treatments, we suggest that autologous PRGFs offer a wider and more natural variety of growth factors, resulting in reduced immune reaction and personalized treatment for patients needs. As PRGF is a serum and cell‐free liquid injectable, its application is not limited to the skin and provides potential for inflamed tendons, synovia, or bone cells similar to PRP. The main benefit of PRGF would be the instant delivery of growth factors to the application site, whereas other platelet products like PRP or platelet‐rich fibrin release their growth factors upon fibrin degradation. As biostimulators are trending in aesthetic medicine, we suggest investigating PRGF in further studies to evaluate its biostimulatory potential.

We are able to show that PRGFs improve keratinocyte cell viability while inducing a terminal differentiation‐like morphology. These findings are in accordance with previously published data by Bayer et al., who used a similar platelet protocol and reported PRGF‐induced upregulation of involucrin and transglutaminase‐2 and keratinocyte differentiation. In addition, Singh et al. observed significant induction of matrix metalloproteinase‐2, semaphorin 7A, angiopoietin‐like 4, and subunit B of PDGF gene expression in keratinocytes stimulated for more than 24 h or exceeding 10% PRGF, indicating cellular involvement in curing hard‐to‐heal wounds [8, 10]. SFN treatment did not influence keratinocyte morphology but increased overall cell viability and proliferation dose‐dependently. Stimulant concentrations exceeding 10 μM SFN and 10% PRGF lead to reduced cell viability and proliferation, indicating overstimulation and cell toxicity.

Next to cellular activation, we were able to provide data on significantly increased mRNA and protein levels of NQO1 and HO‐1 as a result of PRGF and SFN treatment by RT‐qPCR and western blot. These findings were in accordance with previously published data for the treatment of tenocytes and osteocytes with PRGFs, indicating a similar mode of action for keratinocytes [11, 12]. Interestingly, the ARE activity does not decline to the baseline after 48 h of treatment. SFN is an electrophile that rapidly targets the NRF2–KEAP1 interaction; we hypothesize from our experiments that PRGF may activate NRF2 by both known pathways, either via growth‐factor‐receptor‐mediated GSK‐3 blockage or via present electrophiles such as cations, resulting in NRF2 upregulation by MAPK and IPK3 [16, 42].

The anti‐inflammatory effect of PRP and PRGF‐like products has been published by others by investigating different cell types and cutaneous tissue [3, 6, 37, 43]. Coincidentally, PRGF has been shown to decrease oxidative stress and limit neuro‐inflammation in retinal pigment epithelial cells in photodynamic co‐stimulation [4]. Guided by these studies and results, we investigated the effect of PRGF on inflammation‐activated cells using an in vitro keratinocyte NF‐κB‐Luc model. We strictly applied SFN as a positive control for NRF2 activation and NF‐κB downregulation. Our data show that PRGF mediates cellular resilience against the inflammatory stressor TNF‐α, but also that it alleviates TNF‐α‐induced inflammation, shown aa s function of NF‐κB‐Luc signaling within 24 h. Next to the luciferase assay, we could show reduced presence NF‐κB and activated NF‐κB by western blotting. On a functional level, the secretion of inflammatory cytokine TNF‐α was significantly reduced by PRGF and SFN post stimulation. Moreover, we could show that PRGF downregulates TNF‐α induced TNF‐α secretion. The inhibitory effect peaks at 24 h of PRGF treatment, which is in accordance with our ARE‐Luc and NF‐κB‐Luc data, underlining NRF2 involvement. Interestingly, these findings could not be reproduced in the western blot data. SFN did reduce p‐NF‐κB‐p65 and NF‐κB p‐p65, similar to untreated cells, however, single stimulation with PRGF resulted in slightly increased levels for both proteins.

ML‐385 mediated NRF2 inhibition offers fast and reliable NRF2 inhibition in vitro, which is a benefit in the context of the low passaging capacity of patient‐derived primary keratinocytes. This chemical inhibition allowed us to investigate the NRF2 associated ARE and NF‐κB pathways and functional effects transiently without causing genetically induced alterations to the cell. Conversely, NRF2‐inhibitory cell‐culture mediated by ML‐385 treatment reversed both ARE and NF‐κB signaling in our study. SFN is reportedly a strong inhibitor of NF‐κB and its downstream processes in multiple cells and tissues, such as corneal cells, synovial fibroblasts, keratinocytes, and dendritic cells, that can be directly referenced to NRF2 activation and NRF2 translocation [44, 45]. In accordance with this, we were able to demonstrate HO‐1 upregulation in PRGF and SFN stimulated keratinocytes, which is a well‐described NRF2‐associated inhibitor of the NF‐κB‐p65 pathway [46]. Based on our data, the interaction between PRGF and NRF2‐ARE appears to clear inflammatory conditions through a mechanism similar to the inhibition of NF‐κB by SFN [47].

In order to investigate the observed anti‐inflammatory and cytokine‐regulative effects on NRF2 availability, we extended our experimental set‐up to ML‐385 mediated NRF2‐inhibitory conditions. We demonstrated that PRGF's influence on the secretion of IL‐1β, IL‐6, IL‐4, and IL‐10 is dependent on ML‐385 treatment and therewith NRF2 availability. The ELISA results for PRGF‐treated keratinocytes were in strong accordance with previously published studies reporting that NRF2 activation directly inhibits NF‐κB and downregulates IL‐1β and IL‐6 [48]. In a previous study, we demonstrated that PRGF reduces inflammation markers such as IL‐1β, IL‐,6 and TNF‐α in chondrocytes, which is in compliance with our results indicating a universalinflammation‐reducingg and cell‐type independent effect of PRGF treatment [49]. Our findings contribute to the beneficial effects observed by PRP treatments used as effectiv, regenerative therapy after aesthetic procedures in the clinic, all of which go along with an initial inflammatory phase [50].

However, limitations for the use of PRGF should be addressed application specific and it should be highlighted that every PRGF is patient specific, resulting in unique composition. While inflamed keratinocytes benefit from PRGF treatment, this might not be the case for pathologic cellular conditions such as cancer or diseases that change platelet function and activity or require precise and selective growth factor treatment. Our results underline the potential of PRGF; however, we recommend further investigating specific inflammatory pathways associated with cytokine regulation to fully characterize its mode of action. Technical limitations of our study should be addressed in association with the low passaging of primary keratinocytes, which renders the generation of stable NRF2 knock‐outs almost impossible in vitro. In vivo, studies have shown that keratinocytes undergo proliferation by NRF2 activation through a fibroblast‐senescent phenotype that results in excessive growth‐factor release or even hyperkeratosis in KEAP1 knock‐out rodents [27, 51]. Further, all cells used in this study came from male donors, so a sex‐specific bias cannot be excluded in regard to NRF2. Lastly, it can be stated that PRGF activates pathways involved in oxidant clearance, cytoprotection, cellular activation, and IL‐regulation that may attenuate skin inflammation and improve dermal properties. Our findings help to add value to the molecular understanding of the observed anti‐inflammatory effect of PRP/PRGF by introducing NRF2 contribution. A highly relevant observation for skin regeneration and future therapies that require local and fast anti‐inflammatory treatment and rapid oxidant clearance, such as dermatological needling or laser treatment [51, 52, 53]. In this context, NRF2 activators have already been suggested as possible treatment options for both inflammation and regeneration similar to stem‐cell therapy [26, 53, 54]. We suggest further investigation of our serum‐free PRP lysate PRGF in vitro or in vivo to target regenerative and rejuvenating cellular markers in the clinic.

## Author Contributions

**Mersedeh Tohidnezhad, Hannes Klump, Jens Baron, and Thomas Pufe:** conceptualization. **Matthias Stein and Thomas Pufe:** data curation. **Matthias Stein:** formal analysis. **Matthias Stein:** investigation. **Matthias Stein, Nicole Böttcher, Athanassios Fragoulis, Andreas Bayer, Hannes Klump, and Jens Baron:** methodology. **Hannes Klump, Jens Baron, and Thomas Pufe:** resources. **Mersedeh Tohidnezhad and Thomas Pufe:** supervision. **Matthias Stein:** writing – original draft. **Nicole Böttcher, Mersedeh Tohidnezhad, Athanassios Fragoulis, Andreas Bayer, Hannes Klump, Jens Baron, and Thomas Pufe:** writing – review and editing.

## Consent

The authors have nothing to report.

## Conflicts of Interest

The authors declare no conflicts of interest.

## Supporting information


Data S1.

## Data Availability

The original contributions presented in the study are included in the article/Data S1; further inquiries can be directed to the corresponding author.
