# Supplementary material for: Platelet‐Released Growth Factors (PRGFs) Activate NRF2‐ARE and Modulate Inflammatory Response in an NRF2‐Dependent Manner in Primary Human Keratinocytes
Source: J Cosmet Dermatol. 2025 May 12;24(5):e70228. doi: 10.1111/jocd.70228 (PMC12067853; doi:10.1111/jocd.70228)
Supplement: Supplementary file 1 — Data S1. [file JOCD-24-e70228-s001.pdf]

Figure S1 Updated with TNF-a

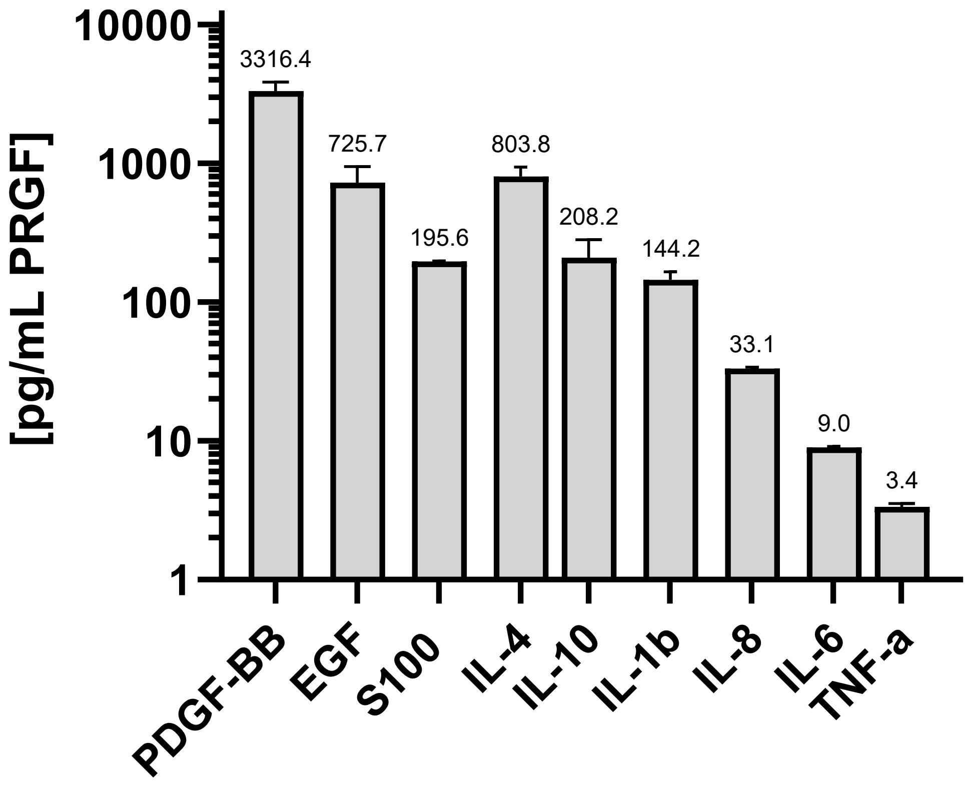

Figure S2: Selected Reference Genes

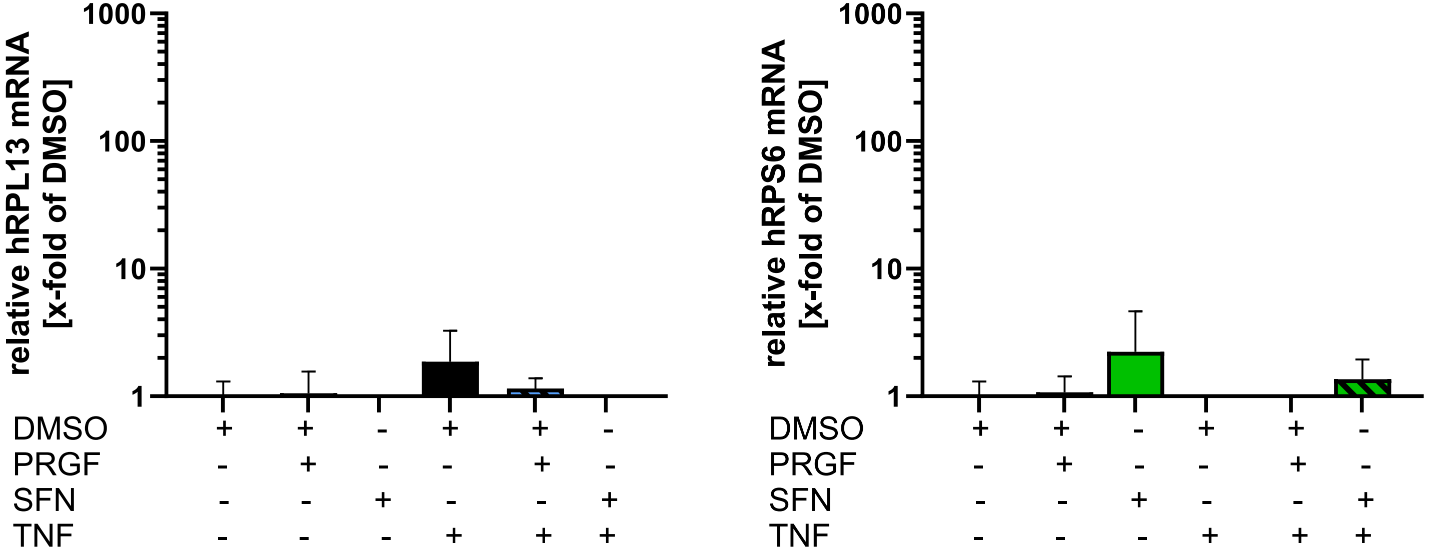

Figure S3: Donor Information

Label	Sex	Age	Source	Initial Passage	Extraction
**K241**	m	4	foreskin	0	2005
**K304**	m	17	foreskin	0	2007
**K252**	m	2	foreskin	0	2005
**K232**	m	5	foreskin	1	2014
**K231**	m	x	foreskin	0	2014
**K312**	m	8	foreskin	1	2007
